# Distribution and quality of privately-funded general practices in England: a cross-sectional analysis

**DOI:** 10.3399/BJGPO.2025.0116

**Published:** 2026-01-28

**Authors:** Joseph Hutchinson, Michael Anderson, Harriet Bullen, Zara Kurdo, Matt Sutton

**Affiliations:** 1 National Centre for Sport and Exercise Medicine, Loughborough University, Loughborough, UK; 2 Centre for Primary Care and Health Services Research, The University of Manchester, Manchester, UK; 3 LSE Health, London School of Economics and Political Science, London, UK

**Keywords:** accessibility, general practice, private healthcare sector, primary health care, quality of health care, socioeconomic status

## Abstract

**Background:**

Public surveys indicate demand for privately funded general practice services in England has increased. However, little is known regarding the number, location, and quality of private general practices.

**Aim:**

To examine: (1) the geographical distribution of private general practices across England; (2) the relationships between access, continuity of care, and funding of NHS general practices with nearby private practices; and (3) the quality ratings of NHS and private general practices.

**Design & setting:**

Cross-sectional analysis of NHS and private general practices in England as of April 2024.

**Method:**

We used the Care Quality Commission (CQC) Primary Medical Services Inspectorate to identify all private general practices in England. We used multilevel logistic regression to examine associations between NHS practice characteristics and the presence of a private general practice nearby. We then compared available CQC ratings.

**Results:**

As of April 2024, England had 358 private and 5976 NHS practices. Private general practices are primarily in London and other urban areas. NHS practices with higher patient satisfaction with waits for appointments (odds ratio [OR] 1.08 [95% confidence interval {CI} = 1.03 to 1.13]) and more GPs per 10 000 patients (OR 1.04 [95% CI = 1.01 to 1.06]) were more likely to have a private practice nearby. There was no association with continuity of care or funding. Quality ratings were similar, although 43.6% of private practices were unrated by the CQC.

**Conclusion:**

Private general practices are more common in London, as well as areas with better access to NHS GPs. The growth in private general practices may have widened inequalities in access to primary care.

## How this fits in

There are reports that private general practice is growing in the UK, but there is little published research. We find provision of private general practice is concentrated in London and areas that already benefit from better access to NHS GPs. The growth of private general practices may increase inequality in access to primary care. Quality is largely unknown, with 43.6% of private general practices open for over a year not yet receiving inspection.

## Introduction

Since the establishment of the NHS, some privately funded care has been used to access services more quickly, or types of care not funded by the NHS.^1,2^ Most private health care has taken place outside of general practice^3,4^ as, relative to other high-income countries, UK citizens have historically enjoyed good access to universal, comprehensive, and high-quality primary care.^5,6^ However, there are growing difficulties in accessing NHS general practice owing to rising demand and fewer GPs.^7^ Satisfaction with NHS general practice services declined from 68% to 34% between 2019 and 2023, with 34% of patients now feeling wait times are too long.^8,9^

One-third of the UK population are now accessing privately funded health care, with 24% of them accessing private general practice.^10^ There are also intergenerational differences, with 45% of 18–24 year olds that use privately funded care using private general practice.^10^ In response, several companies have emerged.^11^ An estimated 2000–3000 GPs provide privately funded care in the UK, with a market that was initially focused on London.^12^

Despite this growth, there has been no systematic analysis of the distribution of private general practices across England. Understanding their location is necessary to understanding equity of access to primary care.^13^

In the hospital sector, there are concerns about the quality and safety of private providers.^14–18^ Similar concerns exist in the long-term care setting, where for-profit ownership of care homes is associated with lower quality.^19^ It is therefore important to compare the quality of care between NHS and private practices.

This paper addresses these gaps in three ways. First, by mapping the location of private general practices in England. Second, by examining associations between measures of access, continuity of care, and funding in NHS practices and the presence of nearby private general practices. Third, by comparing quality measures between NHS and private practices.

## Method

### Study design

A cross-sectional analysis of all NHS and private general practices in England as of the 3 April 2024.

### Care Quality Commission data

The Care Quality Commission (CQC) is the independent regulator of all health and social care organisations in England. All healthcare organisations must register with the CQC.^15^ Part of this regulation are inspections in which organisations are rated inadequate, requires improvement, good, or outstanding.^20^ We gathered the ratings, postcode, and date of last inspection for all organisations registered with the CQC Primary Medical Services Inspectorate as of 3 April 2024.^21^

We classified organisations into the following three types: a private general practice; other private outpatient service; or other service provider. We defined a private general practice as one providing privately funded care, and comprehensive and first point of contact care with a GP, meaning we judged that you could consult without restrictions.^22^ Private outpatient services also provided privately funded care, encompassing a broad range of activity such as menopause-only clinics. Organisations were classified as ‘other service providers’ if they only provided NHS services, devices, dentistry, pharmacy services, or estates. NHS practices were defined as practices that held an NHS contract to deliver NHS-funded primary care services.

We excluded ‘other service providers’ and organisations with insufficient information to categorise. We excluded private outpatient services from the main analysis, but performed a supplementary analysis. We categorised organisations as either physical or remote-only, with remote-only providers being excluded as our analysis relied on geographical location.

This classification process was conducted by ZK (73%) and JH (27%) reviewing relevant websites. Any uncertainty in classification was discussed, with MA acting as a third reviewer to decide the final classification and ensure consistency.

The CQC aims to assess new organisations within 12 months of registration.^20^ Owing to the low numbers of private general practices and other private outpatient services that have been rated, we could not compare the individual domains as our models require 10 events per predictor.^23^ As such, we created a binary indicator of whether an organisation had received a requires improvement or inadequate rating in any CQC domain (that is, Safe, Effective, Caring, Responsive, Well-Led, and Overall).

### Additional data

#### NHS Organisational Data Service

A full list of active NHS general practices was gathered from NHS England’s Organisational Data Service.^24^

#### Area-level demographics

Data on which patients use private general practices is not available. Instead, we linked the organisation’s postcode to the corresponding Middle level Super Output Area (MSOA).^25^ MSOAs comprise of 5000–15 000 people. We then gathered census data for each MSOA, including age (percentage of patients aged <14 years, 14–44 years, 45–64 years, and ≥65 years)^26^ sex (percentage of male patients)^25^ and ethnicity (percentage ethnicity other than White),^27^ 2019 Index of Multiple Deprivation decile (10 = least deprived),^28^ rurality (urban or rural),^29^ and NHS integrated care board (ICB). ICBs are regional organisations in England responsible for planning and commissioning health services for local populations, ranging between 500 000 and 3 million people.^30^

#### NHS practice factors

We collated information on NHS practice factors that may influence the presence of nearby private practices. This included the following: practice list size;^31^ age (percentage aged ≤14 years, 15–44 years, 45–64 years, and ≥65 years);^31^ sex and ethnicity of registered patients; payment per registered patient;^32^ patient satisfaction with access and continuity (access was based on the question, *‘How do you feel about how long you waited for your appointment?’* [percentage about right], and continuity was based on the question, *‘How often do you get to see or speak to your preferred healthcare professional when you ask to?’* [percentage always or almost always, a lot of the time {total}] from the 2024 GP Patient Survey);^33^ clinical quality (from the 2022–2023 total Quality and Outcomes Framework [QOF] points);^34^ and workforce (was based on full-time GPs per 10 000 patients).^31^

### Presence of nearby private general practices

Using the longitude and latitude for each practice postcode, we identified the nearest 10 general practices (mean 7.58 km). We used Euclidean distance, which is the most common approach when measuring how distance impacts access.^35^ We then created a binary variable if any of these 10 practices were privately funded. We repeated this process for the other outpatient organisations. This analysis allows for variations in population density, which is important as rural residents will travel further for care.^35^ We also performed a sensitivity analysis of the nearest 20 organisations (mean 11.00 km).

### Data quality

In total, 43.6% of private general practices had missing CQC ratings, which we categorised separately as ‘not rated’. The proportion of the practice list of an ethnicity other than White had the greatest missing data (0.50%), while 99% of observations had full data availability.

### Data analysis

Data analysis was conducted in R (version 4.3.3). Initial descriptive analyses considered the practice opening date, and their geographic and demographic distribution.

#### Presence of nearby private general practice

We conducted multilevel logistic regression for the presence of a nearby private practice within the 10 closest organisations, including practice and area-level characteristics at level 1 and ICB at level 2. We adjusted for ethnicity and age, rurality, deprivation, and clinical quality.

Sensitivity analyses were conducted excluding London practices, with other outpatient services and considering the nearest 20 organisations.

#### CQC ratings

First, we performed single-level logistic regression to compare whether private practices open for at least 12 months were more likely to have received a CQC rating compared with NHS practices. This was also repeated for other private outpatient services. Second, we compared whether private organisations, relative to NHS general practices, were more likely to have a requires improvement or inadequate rating in any CQC rating category. To ensure robustness of the approach, we compared the ratings of NHS practices with those of both private general practices and other outpatient services combined. We adjusted for ethnicity, age, deprivation, and rurality.

## Results

### Descriptive statistics

Our dataset included 5976 NHS practices, 358 private general practices, and 582 other private outpatient services. Regarding location, 50.6% of private general practices were based in London, compared with 18.0% of NHS practices (Table 1). While 0.28% of private general practices were located in the North East versus 5.1% of NHS practices. Regarding rurality, 95.5% of private general practices were in urban areas, compared with 84.3% of NHS practices. The mean deprivation decile was 6.5 (95% confidence interval [CI] = 6.2 to 6.7) for private practices and 5.0 (95% CI = 4.9 to 5.0) for NHS practices, meaning private general practices were located in more affluent areas. They are also located in areas with more ethnic minorities.

**Table 1.** Descriptive statistics of the variables used for the CQC models (full breakdown of CQC rating criteria in supplementary material) as well as the region, full age profile, and weeks since the last CQC rating as of 3 April 2024

Characteristic	NHS practice *n* = 5976	95% **CI**	Other private outpatient service *n* = 582	95% **CI**	Private general practice *n* = 358	95% **CI**
Proportion MSOA aged <14 years, mean (SD)	0.16 (0.04)	0.16 to 0.16	0.13 (0.05)	0.12 to 0.13	0.13 (0.05)	0.13 to 0.14
Proportion MSOA aged 14–44 years, mean (SD)	0.41 (0.09)	0.40 to 0.41	0.46 (0.13)	0.45 to 0.47	0.47 (0.13)	0.46 to 0.49
Proportion MSOA aged 45–64 years, mean (SD)	0.25 (0.04)	0.25 to 0.25	0.25 (0.05)	0.24 to 0.25	0.24 (0.05)	0.24 to 0.25
Proportion MSOA aged ≥65 years, mean (SD)	0.18 (0.07)	0.18 to 0.18	0.16 (0.07)	0.15 to 0.17	0.15 (0.07)	0.15 to 0.16
Proportion MSOA ethnicity other than White, mean (SD)	0.31 (0.26)	0.30 to 0.31	0.45 (0.26)	0.43 to 0.47	0.49 (0.24)	0.46 to 0.52
Proportion MSOA male, mean (SD)	0.49 (0.02)	0.49 to 0.49	0.49 (0.02)	0.49 to 0.50	0.49 (0.03)	0.49 to 0.49
IMD decile, mean (SD)	4.95 (2.87)	4.9 to 5.0	6.63 (2.61)	6.4 to 6.8	6.45 (2.65)	6.2 to 6.7
**Rurality*,* n (%)**						
Rural	937 (15.68)	15 to 17	58 (9.97)	7.7 to 13	16 (4.47)	2.7 to 7.3
Urban	5039 (84.32)	83 to 85	524 (90.03)	87 to 92	342 (95.53)	93 to 97
**Region, n (%)**						
East Midlands	495 (8.28)	7.6 to 9.0	26 (4.47)	3.0 to 6.6	11 (3.07)	1.6 to 5.6
East of England	585 (9.79)	9.1 to 11	45 (7.73)	5.8 to 10	29 (8.10)	5.6 to 12
London	1078 (18.04)	17 to 19	256 (43.99)	40 to 48	181 (50.56)	45 to 56
North East	304 (5.09)	4.6 to 5.7	10 (1.72)	0.88 to 3.2	1 (0.28)	0.01 to 1.8
North West	926 (15.50)	15 to 16	49 (8.42)	6.4 to 11	32 (8.94)	6.3 to 13
South East	791 (13.24)	12 to 14	98 (16.84)	14 to 20	54 (15.08)	12 to 19
South West	510 (8.53)	7.8 to 9.3	43 (7.39)	5.5 to 9.9	15 (4.19)	2.4 to 7.0
West Midlands	717 (12.00)	11 to 13	29 (4.98)	3.4 to 7.2	20 (5.59)	3.5 to 8.6
Yorkshire and The Humber	570 (9.54)	8.8 to 10	26 (4.47)	3.0 to 6.6	15 (4.19)	2.4 to 7.0
Weeks from last CQC rating, mean (SD)	271.44 (130.90)	268 to 275	118.30 (75.05)	109 to 127	143.30 (79.75)	132 to 154

CI = confidence interval. CQC = Care Quality Commission. IMD = Index of Multiple Deprivation. MSOA = Middle layer Super Output Area

Regarding registration, 82.7% and 100% of private and NHS practices were registered with the CQC at least 12 months before the 1 April 2024. Of practices open in April 2024, 10 private and five NHS practices were established in 2013–2014, while 67 private and one NHS practice opened in 2022–2023 (Supplementary figure S4).

**Figure 1.**
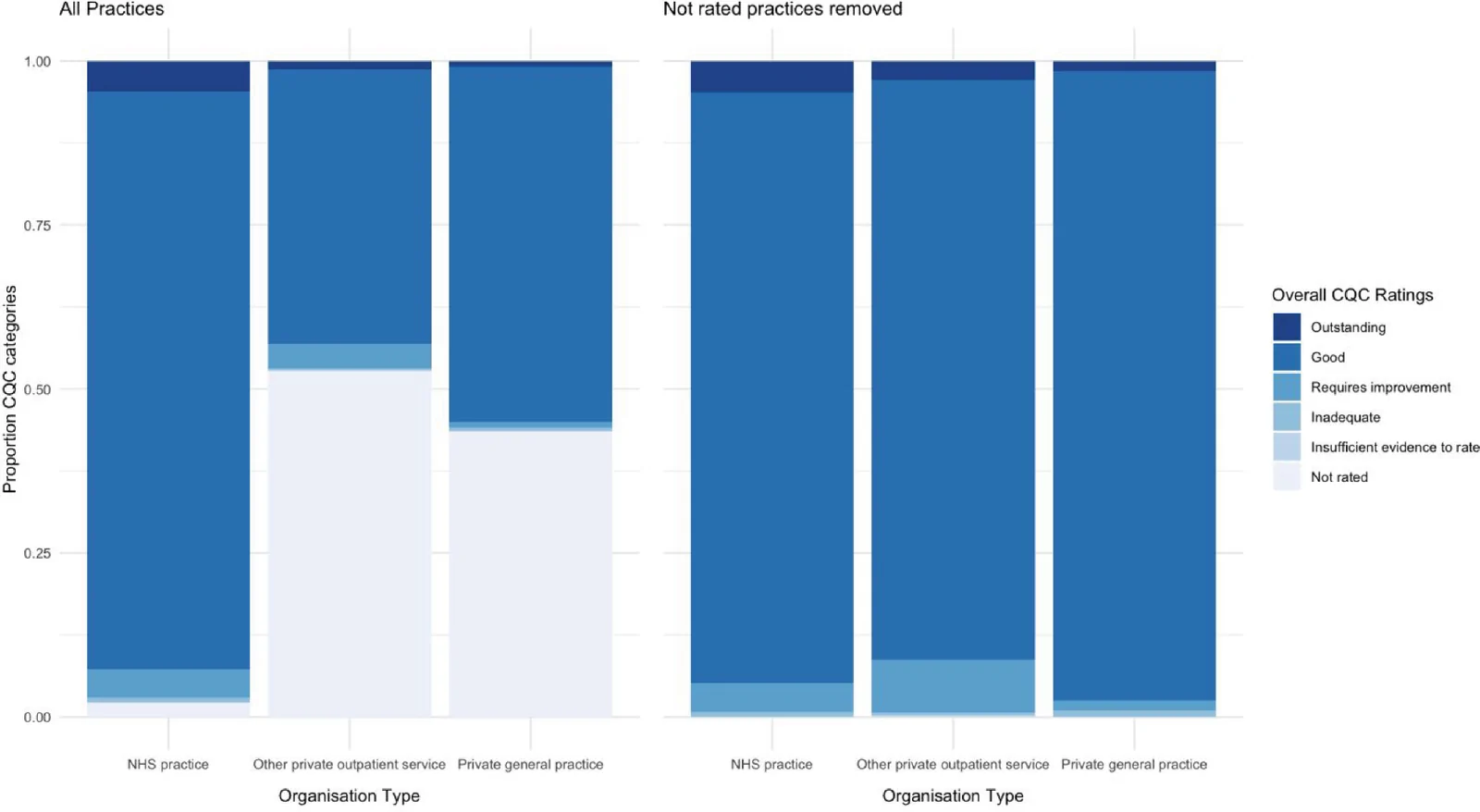
Stacked bar chart of CQC ratings by organisation type, with and without ‘not rated’ practices. CQC = Care Quality Commission

Among practices open at least 12 months, 43.6% of private and 2.2% of NHS practices were not rated by the CQC (Supplementary table S2 and Figure 1). If rated, mean time since last review was 271 and 143 weeks for NHS and private practices, respectively.

### Main analysis

In total, 29.4% of NHS practices had a private general practice within the nearest 10 practices (Table 2). Patient satisfaction with the waiting time for NHS appointment (odds ratio [OR] 1.08 [95% CI = 1.03 to 1.13]) and the number of FTE GPs per 10 000 patients (OR 1.04 [95% CI = 1.01 to 1.06]) were positively associated with the presence of a private practice nearby (Table 3). A 10% increase in satisfaction with wait times was associated with 1.08 times higher odds of a private general practice nearby. Each additional FTE GP per 10 000 patients was associated with 1.04 times increased odds. Neither patient satisfaction with continuity of care (OR 1.01 [95% CI = 0.97 to 1.05]) nor payments per patient (OR 1.02 [95% CI = 1.00 to 1.03]) to NHS practices were statistically significant.

**Table 2.** Descriptive statistics of the variables used in the models analysing private and other private outpatient services surrounding NHS practices

Characteristic	*n* = 5936	95% **CI**
Wait for appointment (proportion about right), mean (SD)	0.68 (0.14)	0.67 to 0.68
GPs per 10 000 patients, mean (SD)	5.83 (2.61)	5.8 to 5.9
Preferred healthcare professional (proportion always or almost always, a lot of the time [total]), mean (SD)	0.41 (0.18)	0.41 to 0.42
Average payment per registered patient, mean (SD)	167.13 (50.68)	166 to 168
Adjacent private general practice, *n* (%)	1746 (29.41)	28 to 31
Adjacent other private outpatient service, *n* (%)	2372 (39.96)	39 to 41
IMD decile, mean (SD)	4.96 (2.87)	4.9 to 5.0
**Rurality, *n* (%)**		
Rural	933 (15.72)	15 to 17
Urban	5003 (84.28)	83 to 85
QoF score (total points), mean (SD)	575.79 (50.65)	575 to 577
Proportion practice list male sex, mean (SD)	0.50 (0.02)	0.50 to 0.50
Proportion practice list aged ≤14 years, mean (SD)	0.16 (0.03)	0.16 to 0.16
Proportion practice list aged 15–44 years, mean (SD)	0.40 (0.09)	0.40 to 0.40
Proportion practice list aged 45–64 years, mean (SD)	0.25 (0.04)	0.25 to 0.26
Proportion practice list aged ≥65 years, mean (SD)	0.18 (0.07)	0.18 to 0.18
Proportion practice list ethnicity other than White	0.20 (0.21)	0.20 to 0.21

CI = confidence interval. IMD = Index of Multiple Deprivation. QoF = Quality and Outcomes Framework

**Table 3.** Results of multilevel logistic model for whether an NHS practice has a private GP within their 10 closest organisations of NHS and private general practices

	Model 1: Satisfaction with wait for appointment		Model 2: Full-time GPs per 10 000 patients		Model 3: Satisfaction with continuity of care		Model 4: Payment per patient	
	**OR**	**95% CI**	**OR**	**95% CI**	**OR**	**95% CI**	**OR**	**95% CI**
Satisfaction with appointment wait	1.08^a^	1.03 to 1.13						
FTE GPs per 10 000 patients			1.04^a^	1.01 to 1.06				
Satisfaction with continuity					1.01	0.97 to 1.05		
Payment per patient							1.02	1.00 to 1.03
**Covariates**								
Proportion practice list ethnicity other than White	1.05	0.99 to 1.11	1.04	0.98 to 1.09	1.04	0.98 to 1.09	1.03	0.98 to 1.09
Proportion practice list aged <14 years	0.52^a^	0.43 to 0.65	0.51^a^	0.42 to 0.63	0.51^a^	0.41 to 0.62	0.51^a^	0.41 to 0.63
Proportion practice list aged >65 years	0.60^a^	0.52 to 0.70	0.58^a^	0.51 to 0.68	0.60^a^	0.52 to 0.70	0.58^a^	0.50 to 0.67
Rurality (urban)	1.48^a^	1.19 to 1.84	1.46^a^	1.18 to 1.81	1.45^a^	1.17 to 1.80	1.61^a^	1.26 to 2.05
Community IMD decile	1.13^a^	1.10 to 1.17	1.14^a^	1.10 to 1.17	1.13^a^	1.10 to 1.17	1.14^a^	1.11 to 1.17
QoF score	0.99	0.98 to 1.00	0.99	0.98 to 1.00	0.99	0.98 to 1.01	0.99	0.98 to 1.01
**Random effects (integrated care board)**								
Level 1 variance	3.29		3.29		3.29		3.29	
Level 2 variance	0.99		0.99		0.98		0.99	
Variance partition coefficient, %	23.13		23.13		22.95		23.13	

^a^ *P*-value <0.05. CI = confidence interval. FTE = full-time equivalent. IMD = Index of Multiple Deprivation. OR = odds ratio. QoF = Quality and Outcomes Framework

QoF score estimates the impact from an additional 10 QoF points. GPs per 10 000 patients details the impact of another GP. Payment per patient details the impact of another £10. Meanwhile the other continuous variables estimate the impact of a 10% increase.

**Figure 2.**
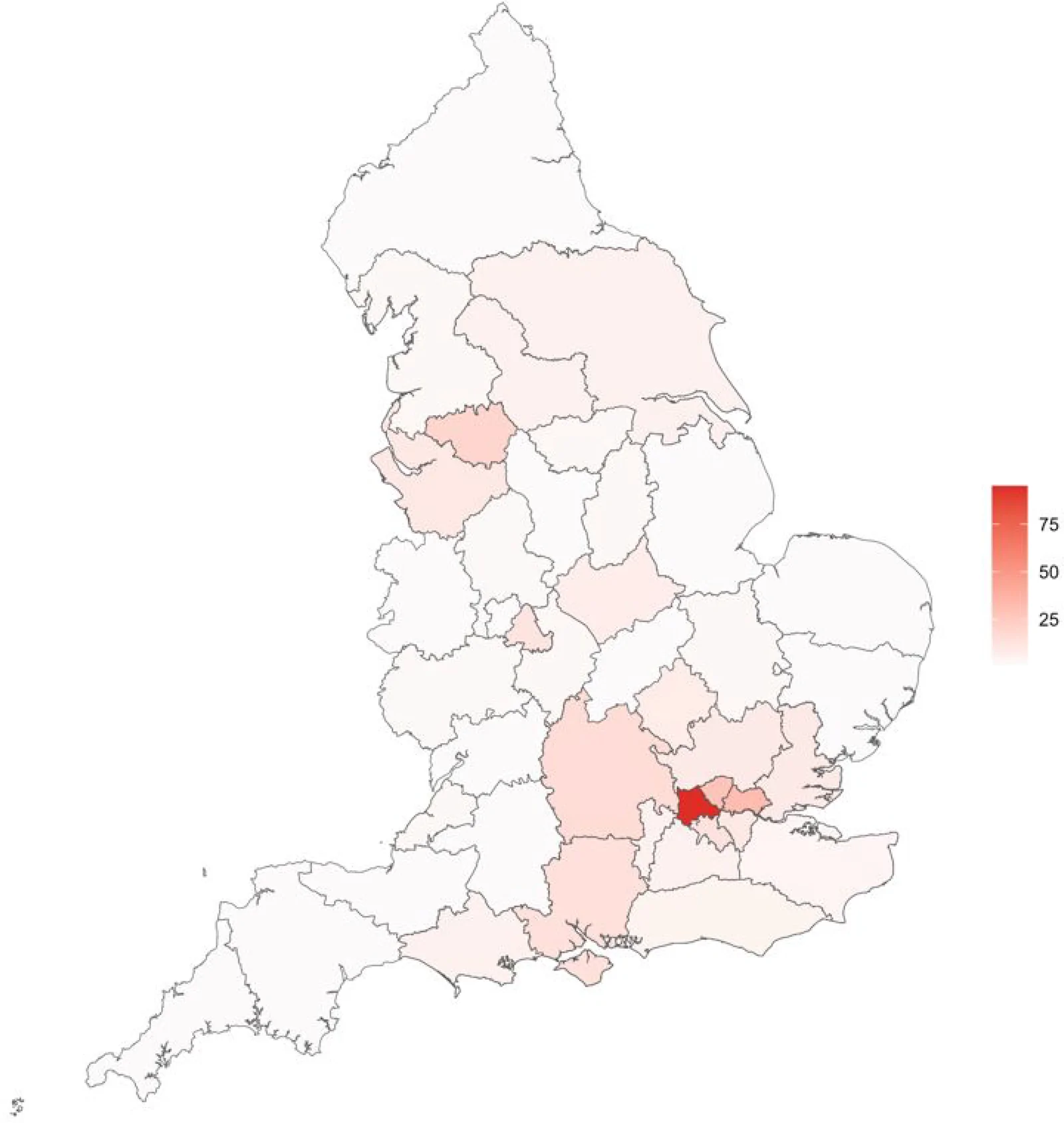
Choropleth of private general practices by integrated care board in England

Nearly one-quarter (23%) of the variation in presence of private practices was owing to the clustering within ICBs (Supplementary figure S1). Generally, South East and London ICBs had more private practices compared with the rest of England (Figure 2). However, there are some exceptions. For example, NHS Kent and Medway had lower and NHS Humber and North Yorkshire higher.

Caution is needed when interpreting the covariates. However, urban areas, less deprivation, and a greater proportion of patients aged between 14 years and 64 years were associated with higher likelihood of private practices.

Private general practices (OR 0.03 [95% CI = 0.02 to 0.04]) and other private outpatient services (OR 0.03 [95% CI = 0.02 to 0.03]) had lower odds of being rated by the CQC than NHS practices (Table 4). When combining private general practices and other outpatient service, we identified no significant differences in their CQC rating compared with NHS practices.

**Table 4.** Results of single-level logistic regression for whether the private general practice and other private outpatient service has been rated or not, as well as single-level logistic regression model whether NHS practices have different odds of no CQC rating category below good compared with private general practices and other outpatient services combined.

	Model 1: Private general practice		Model 2: Other private outpatient service		Model 3: NHS practice versus private general practice AND other outpatient service: good or outstanding	
	**OR**	**95% CI**	**OR**	**95% CI**	**OR**	**95% CI**
Main variables						
Private general practice	0.03^a^	0.02 to 0.04				
Other outpatient service			0.03^a^	0.02 to 0.03		
NHS practice					0.84	0.62 to 1.15
Covariates						
Proportion MSOA ethnicity other than White	2.65^a^	1.21 to 5.91	1.49	0.75 to 3.01	1.79^a^	1.30 to 2.46
Proportion MSOA list aged <14 years	0.63^a^	0.43 to 0.92	0.82	0.59 to 1.14	1.36^a^	1.09 to 1.71
Proportion MSOA list aged ≥65 years	1.13	0.81 to 1.58	1.22	0.90 to 1.65		
Rurality (urban)	0.84	0.49 to 1.39	1.10	0.71 to 1.68	1.23	0.94 to 1.63
Community IMD decile	1.06	1.00 to 1.12	1.04	0.99 to 1.10	0.95^a^	0.92 to 0.98

^a^ *P*-value <0.05. CI = confidence interval. IMD = Index of Multiple Deprivation. MSOA = Middle layer Super Output Area. OR = Odds ratio.

Private GP and other outpatient services details the odds ratio of being rated compared with an NHS practice. NHS practice details the odds ratio of having all ratings good or outstanding compared with private general practices AND other outpatient services. The continuous fixed effects detail the impact of a 10% increase in that variable.

### Supplementary analyses

For other private outpatient services, neither patient satisfaction with the wait for their appointment (OR 1.04 [95% CI = 0.99 to 1.08]), continuity of care (OR 1.01 [95% CI = 0.97 to 1.04]), or payments per patient (OR 1.00 [95% CI = 0.99 to 1.02]) were significantly associated with their presence. However, the number of FTE GPs per 10 000 patients was positively associated (OR 1.03 [95% CI = 1.01 to 1.05]).

Outside of London satisfaction with the wait for the appointment was not statistically significant (OR 1.05 [95% CI = 0.99 to 1.11]). However, our estimates for the number of FTE GPs per 10 000 patients, continuity, and payments per patient were consistent.

Findings remained robust when considering the 20 closest organisations. However, the estimate for satisfaction with the wait for the appointment increased (OR 1.10 [95% CI = 1.04 to 1.15]).

## Discussion

### Summary

Private general practices exist across England, but are concentrated in urban areas, particularly London and the South East. Higher numbers of GPs in NHS practices and higher satisfaction of patients with waiting times for appointments were associated with the presence of private general practices. However, neither continuity of care nor funding were associated. Regarding CQC ratings, 43.6% of private general practices, which had been open for at least 12 months, had not been rated by the CQC.

### Strengths and limitations

To our knowledge, this is the first study to examine the distribution and quality of private general practices in England. We use large, publicly available datasets on all registered private organisations to provide a comprehensive analysis.

However, we focused our analysis on in-person rather than remote services as we examined geographical location. Future research should examine the implications of remote private services. No data are available on the volume of patients and appointments in private services. Our analysis assumes that most patients will access private practices locally. There is no literature on how far patients travel in England for private care, although patients prioritise geographical location when accessing health care.^36^ Finally, to enable an analysis of CQC ratings, we had to combine private general practices and other outpatient services, as well as ratings across quality domains. This should be repeated when more ratings have been conducted.

### Comparison with existing literature

Private general practices are concentrated in London and wealthier areas, with few in rural communities. Private services were also located in other urban areas in the North West and West Midlands. These findings support the private GP forum’s description that London remains the main hub of private general practice, with growth in other urban settings.^12^

Previously, the main reason for accessing private care was reported to be difficulty accessing NHS care.^10^ Conversely, we found that measures of improved access to NHS general practice was positively associated with private practices. This suggests a positive correlation between supply of NHS and private practices.

The inverse care law describes how patient need for care is inversely related to the availability of care.^37,38^ In theory, the inverse care law grows when market forces increase.^38^ More affluent communities have lower levels of multimorbidity and mental illness,^39–41^ and therefore healthcare needs. Despite this, more affluent areas have both more private practices and NHS practices with better access. This suggests private practices may intensify the inverse care law and subsequent health inequalities, by differentially serving affluent communities. While it can be argued that private practice reduces burden on NHS services, we do not find evidence for this argument. Similar findings have been demonstrated when analysing NHS and private referral rates at practice level.^42^

### Implications for research and practice

Private general practices are increasingly prominent in England. However, our analysis was limited by minimal data, extending findings from secondary care.^13^ Interestingly, this extended to the CQC, which had not inspected 43.6% of private practices that had been open for more than 12 months. This creates barriers to examining these services, with implications for informed patient choice.

Potential regulatory responses could address these information gaps. The CQC needs to become more responsive to greater numbers of private services opening. The CQC is accountable to the Department of Health and Social Care, which should ensure that all newly established private practices are rated within 12 months. ^43^ Minimum requirements for activity and outcome reporting for private practices could be mandated by the government. The Competition and Market Authority (CMA) Private Healthcare Market Investigation Order 2014 created such a mandate for private healthcare organisations providing inpatient services.^44^

General practice is central to addressing health inequalities but requires that resources are distributed equitably.^45^ Private practices are more common in wealthier communities and near NHS practices with greater access to services. Future policy may need to consider how to address this, such as increasing differential funding to NHS practices relative to the size of the private healthcare market.

There is also a need to anticipate potential risks to NHS general practices from private services. Private hospitals in England typically treat younger, more affluent, and less medically complex patients compared with NHS hospitals.^46^ If the same exists within private general practices, NHS practices may become increasingly focused on more medically complex patients. Private services may increase workload within NHS services. For example, patients may be directed back to the NHS for further management of clinical activity not offered in private services.^^47^^ This should be the subject of further research.

## Data

Available on request.
